# Opposite Effects of Low and High Doses of Aβ42 on Electrical Network and Neuronal Excitability in the Rat Prefrontal Cortex

**DOI:** 10.1371/journal.pone.0008366

**Published:** 2009-12-21

**Authors:** Yun Wang, Guangping Zhang, Hongwei Zhou, Amey Barakat, Henry Querfurth

**Affiliations:** Neurology Research, Caritas St. Elizabeth's Medical Center, Tufts Medical School, Boston, Massachusetts, United States of America; Chiba University Center for Forensic Mental Health, Japan

## Abstract

Changes in neuronal synchronization have been found in patients and animal models of Alzheimer's disease (AD). Synchronized behaviors within neuronal networks are important to such complex cognitive processes as working memory. The mechanisms behind these changes are not understood but may involve the action of soluble β-amyloid (Aβ) on electrical networks. In order to determine if Aβ can induce changes in neuronal synchronization, the activities of pyramidal neurons were recorded in rat prefrontal cortical (PFC) slices under calcium-free conditions using multi-neuron patch clamp technique. Electrical network activities and synchronization among neurons were significantly inhibited by low dose Aβ42 (1 nM) and initially by high dose Aβ42 (500 nM). However, prolonged application of high dose Aβ42 resulted in network activation and tonic firing. Underlying these observations, we discovered that prolonged application of low and high doses of Aβ42 induced opposite changes in action potential (AP)-threshold and after-hyperpolarization (AHP) of neurons. Accordingly, low dose Aβ42 significantly increased the AP-threshold and deepened the AHP, making neurons less excitable. In contrast, high dose Aβ42 significantly reduced the AP-threshold and shallowed the AHP, making neurons more excitable. These results support a model that low dose Aβ42 released into the interstitium has a physiologic feedback role to dampen electrical network activity by reducing neuronal excitability. Higher concentrations of Aβ42 over time promote supra-synchronization between individual neurons by increasing their excitability. The latter may disrupt frontal-based cognitive processing and in some cases lead to epileptiform discharges.

## Introduction

Neuronal synchronization at different frequency bands may underlie a variety of cognitive processes, including perception, motor performance, attention, learning and memory [1], [2]. For instance, oscillatory synchronization and synchronized firing in neuronal assemblies play an important role in working memory [3]. Various abnormalities in neuronal synchronization have been found in patients with Alzheimer's Disease (AD) and mild cognitive impairment (MCI) as well as in relevant animal models [4], [5], [6]. The direction of these changes can be opposing to include inhibited neuronal synchronization [4] and supra-synchronization sufficient to provoke seizure activity [5].

The accumulation of soluble beta-amyloid (Aβ), especially Aβ42 [7], [8], in the brain of patients and animal models of AD is associated with impairments of cognition and memory [9], [10], [11]. Aβ levels have also been correlated to disturbances in neuronal synchronization [4], [5], [12]. There is great interest in the notion that neuronal networks are the major initial site of damage in AD and chemical synaptic networks have received the most attention in this regard [13], [14]. Many studies revealed that the accumulation of Aβ inhibits chemical synaptic transmissions [15], [16], [17]. Although this could partially explain the inhibition of neuronal synchronization, the mechanism underlying desynchronization in AD has not been fully understood.

Electrical networks are prominently involved in memory formation [2]. The effects of Aβ on electrical networks have not however been investigated. Our work focused on this neglected topic by using multi-neuron patch clamp technique to record synchronized activities between individual neurons participating in electrical networks of the rat prefrontal cortex (PFC) under calcium-free conditions. The PFC is an association region critical for working memory [18] and is vulnerable to pathological changes and functional impairments early in AD [19], [20]. Aβ42 levels accumulate to high levels in PFC of transgenic AD-like mice [21]. We determined the influence of soluble Aβ42 on neuronal synchronization and electrical network activities in *ex vivo* brain slices. The basis for those changes was further examined by collecting crucial parameters of neuronal excitability–action potential (AP)-threshold and after-hyperpolarization (AHP). We found that low and high doses of Aβ42 induced opposite changes in electrical network activity and in neuronal excitability.

## Results

### Recording Electrical Network Activities and Neuronal Synchronization in the PFC

**Figure 1:**
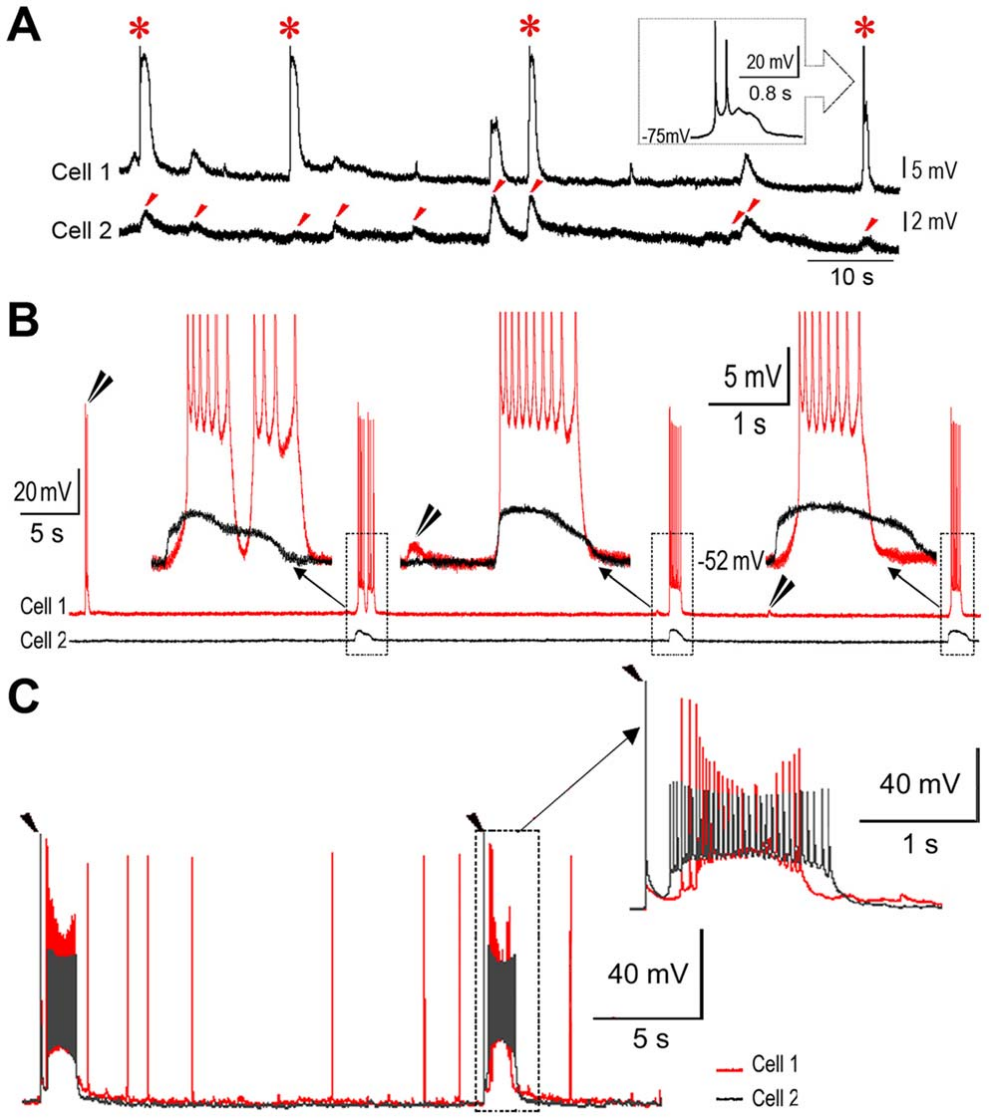
Recordings of synchronized responses between individual neurons in an electrical network of rat PFC. **A**. Synchronized spontaneous depolarizations (marked with red arrow heads). Responses were recorded between two PCs (cell 1 and cell 2) under calcium-free conditions. The depolarizations of cell 1 lead to a few action potentials (APs) (for clarity, APs were truncated as marked with stars). **B**. Synchronized spontaneous bursts and depolarizations. Recording procedure was the same as in **A**. Bursts of cell 1 were synchronized with depolarizations of cell 2. The synchronized responses varied from having equal time courses up to 750 ms differences in duration (as shown in the insets, APs were truncated). Solitary or un-synchronized responses of cell 1 are indicated with double black arrow heads. **C**. Experimentally evoked synchronizing responses. Two neurons (cell 1 and cell2) were simultaneously recorded while two separated extracellular stimuli (20 µA for a duration of 2 ms; marked with single black arrow heads) were delivered locally at 30 s intervals. Bursting responses were provoked in both cells immediately after either of the stimulating artifacts. These bursts were synchronized yet had un-equal durations (see the inset). Following the evoked bursts, spontaneous APs continued to be captured from cell 1, but none from cell 2.

In order to isolate the activity of electrical networks, calcium-free conditions were maintained to inactivate chemical synaptic transmissions [22], [23]. Spontaneous responses of multiple pyramidal cells (PCs) were simultaneously recorded at resting membrane potential using whole-cell patch-clamp technique in the medial PFC of young and adult rats (P16–P30, *n = *108; 3–12 months, *n* = 8). The recorded spontaneous responses included bursts and short depolarizations (from 345 PCs, **Fig. 1A&B**). These responses remained intact when adding blockers of chemical synaptic transmissions (100 µM APV+10 µM DNQX+20 µM picrotoxin, *n = *4, **Fig. S1**). Synchronized responses were obtained in 6% of simultaneously recorded individual neuron pairs (*n* = 11/183 pairs). They varied from having an equal time course to a 750 ms difference in duration (1.8±0.6 s. *n = *11, **Fig. 1B** **insets**). There were no detectable changes in the individual threshold voltages for APs during these recordings, which were all made under calcium-free conditions (see also ref, [24]).

Notable differences in the durations of spontaneous synchronized responses suggested that synchronization among neurons was initiated by a local change in field potential [25], [26]. To test this, a brief extracellular stimulus (10–100 µA for 2 ms) was delivered near simultaneously recorded neurons to trigger a local field potential [25]. As expected, synchronized bursting responses between neurons were experimentally produced (**Fig. 1C**), which had unequal durations (**Fig. 1C** **inset**). To dissect the induction of a provoked burst, a subthreshold depolarization, akin to a field potential experienced by a single neuron, was produced immediately after a weaker extracellular stimulus. Increasing the extracellular stimulus provoked a burst of APs (**Fig. S2**, also see [27]). These experimentally evoked bursts or depolarizations were obtained in 85% of recorded neurons (*n = *80/94) and they were synchronized in 63% of simultaneously recorded pyramidal pairs (n = 38/60 pairs).

**Figure 2:**
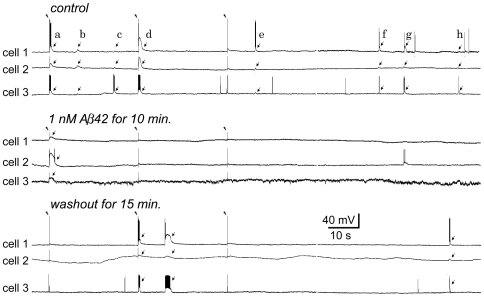
Low dose Aβ42 inhibits synchronized responses in a triple-neuron recording. Under calcium-free conditions, three PCs were simultaneously recorded (cells 1–3). Three extracellular stimuli (30 µA, 2 ms duration) were delivered locally at 30 s intervals (marked with arrow heads). Thereafter the recording was continued for more than 100 s to capture spontaneous responses. This recording procedure was repeated for the conditions of pre-application, application and washout of Aβ. **Upper panel:** In the control condition, 2 (the 1^st^ and 2^nd^) evoked responses (arrows *a* and *d*) and 6 spontaneous responses (arrows *b,c, e–h*) were synchronized between the triple-neuron assembly. The synchronized responses consist of bursts and depolarizations in cell 1 & cell 3 and only depolarizations in cell 2. Note that not all responses were synchronized. **Middle panel:** After 1 nM Aβ42 was applied for 10 min, both evoked and spontaneous responses were notably inhibited. Only 1 subthreshold response was evoked and synchronized among the three cells. Further, burst firing was lost in cell 1 & cell 3. **Lower panel:** After washout of Aβ42 for 15 min., the responses (1 evoked and 2 spontaneous) and their synchronicity tended to recover. Burst firing was also resumed in cells 1 and cell 3.

In the course of a 100 s recording, the percentage (33%) of cells that had spontaneous responses was almost doubled (65%) after three brief extracellular stimuli at 30 s intervals (n = 49, Chi-square test: *p* = 0.001). The enhanced responses included short depolarizations, bursts and tonic firing [26] (**Table S1**). Spontaneous responses were also found to be synchronized more frequently among recorded neurons (**Fig. 2**, upper trace). During the time course of delivering three brief extracellular stimuli at 30 s intervals and extended recording for 100 s, synchronized spontaneous responses appeared in 35% of neuron pairs (n = 21/60 pairs), as well as in triple-cell assemblies (*n = *7). This percentage of post stimulus evoked synchronized spontaneous responses was significantly higher than the basal rate of such occurrences (6%) in the absence of any extracellular stimulus (Chi-square test: *p*<0.0001). These findings indicate that brief extracellular stimuli can prime a local electrical network to produce synchronized activities between individual neurons.

The bursts, short depolarizations and their synchronization represent the activities of electrical networks in the absence of chemical synaptic transmissions [26]. The elicitation of these evoked responses could be reliably controlled by adjusting the strength of extracellular stimuli, making it feasible to examine effects of Aβ42 on neuronal synchronization and electrical network activities below.

### Opposite Effects of Low and High Doses of Aβ42 on Electrical Network Activities

We next examined the effect of Aβ42 peptide on bursts and depolarizations and on their synchronization within electrical networks. Under calcium-free conditions, pairs or triple neuron assemblies were simultaneously recorded while three extracellular stimuli (10–100 µA for 2 ms) were delivered locally at 30 s intervals as above. Thereafter, the recording was extended for more than 100 s to capture more spontaneous responses. This recording procedure was repeated under three conditions: *a)* pre-application, *b)* application and *c)* washout of Aβ42. In a representative experiment, the evoked and spontaneous responses that were frequently synchronized in the control condition (**Fig. 2** **, upper**), were notably inhibited after application of 1 nM Aβ42 for 10 min (**Fig. 2** **, middle**). These responses and their synchronicity tended to recover on washout of Aβ42 for 15 min. (**Fig. 2** **, lower**). Similar phenomena were observed in the recordings from 5 other pairs and another triplet of neurons.

**Figure 3 pone-0008366-g003:**
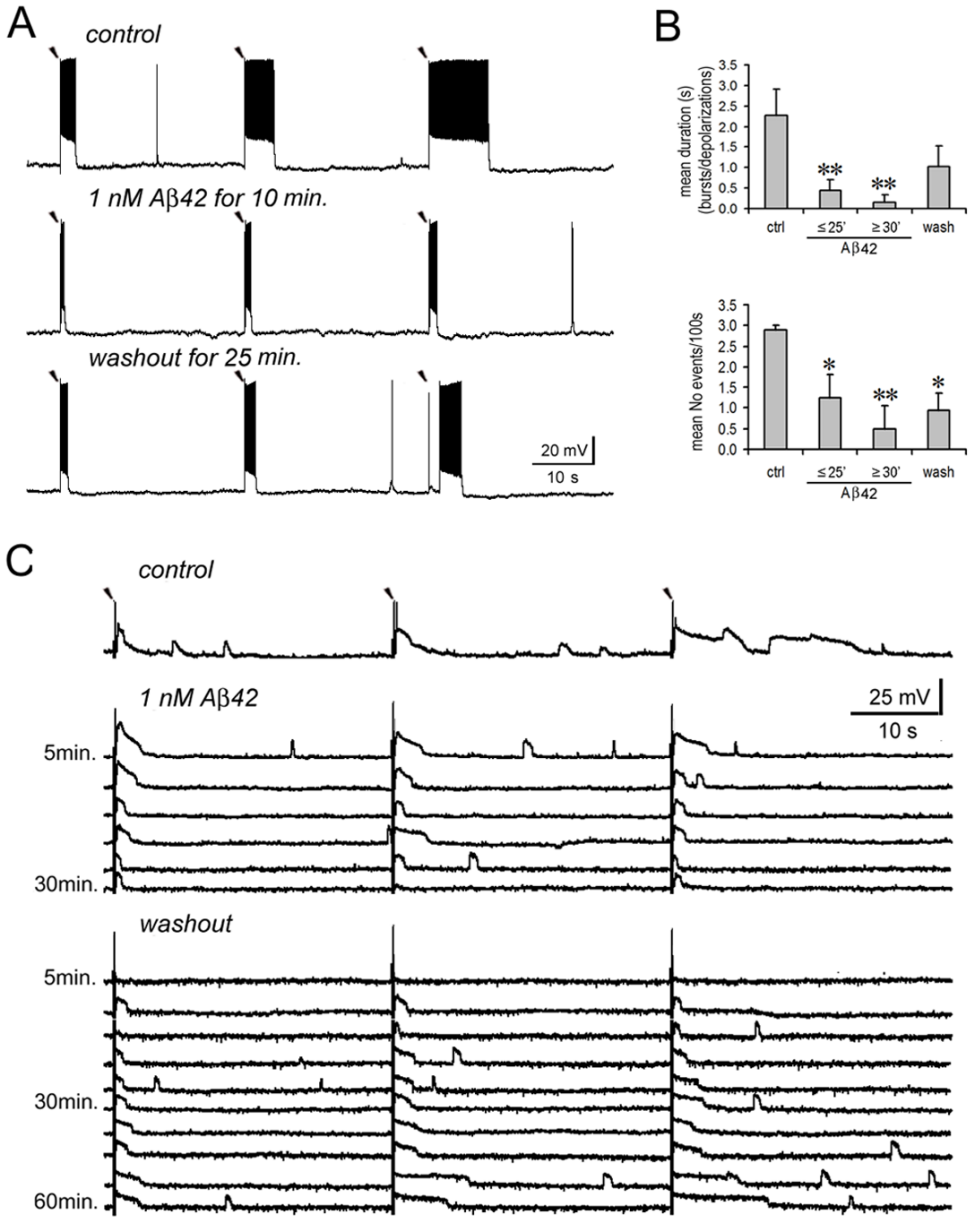
Low dose Aβ42 inhibits electrical network activities. **A.** A representative experiment. Under calcium-free conditions, a PC was recorded while three extracellular stimuli (35 µA, 2 ms duration) were delivered in close proximity. The same recording procedure was repeated in pre-application, application and washout of Aβ. Following each stimulating artifact (marked with an arrow head), burst firing was provoked (upper panel). The bursts became briefer after 1 nM Aβ42 was applied (middle panel), and tended to recover on washout for 25 min. (lower panel). Note that the 3^rd^ evoked burst in washout procedure occurred with a few seconds delay after the stimulating artifact. **B.** Statistical analysis. **Upper panel:** Compared with the control, the average duration of evoked responses was significantly reduced after 1 nM Aβ42 was applied for ≤25 min. (*p* = 0.010, n = 16), and appeared to be further reduced after prolonged applications for ≥30 min. (*p* = 0.009). On washout, the average duration recovered (Difference became insignificant compared with control, *p* = 0.07). **Lower panel:** Compared with the control, the average occurrence of evoked responses was significantly reduced after 1 nM Aβ42 was applied for ≤25 min. (*p* = 0.014), and even further reduced after the application for ≥30 min. (*p* = 0.009). On washout, this occurrence tended to recover but was still significantly lower than the control (*p* = 0.011). **C.** Another representative experiment. The experimental procedure is the same as in **A** (The extracellular stimulus was 30 µA for 2 ms; marked with arrow heads). In the control conditions, evoked and spontaneous depolarizations were visible following each stimulating artifact. After applying 1 nM Aβ42, the same recording was repeated for 30 min. in 5 min. intervals. Over time, evoked depolarizations gradually became narrower, and finally both evoked and spontaneous depolarizations vanished. Following washout, evoked depolarizations re-occurred and became gradually broader. Spontaneous depolarizations also reappeared and became more and more frequent. Note: “*”, *p*<0.05; “**”, *p*<0.01.

The duration and occurrence of evoked bursts and depolarizations were quantified according to different applied concentrations of Aβ42. 1 nM Aβ42 significantly inhibited evoked responses, which became briefer (Aβ for ≤25 min.: 0.4±0.3 s vs. control: 2.3±0.7 s., *p* = 0.010, *n* = 16) and occurred less frequently (Aβ for ≤25 min.: 1.3±0.6 events/100 s vs. control: 2.9±0.1 events/100 s, *p* = 0.014) (**Fig. 3**). The inhibition remained and tended to be strengthened when 1 nM Aβ42 was applied for more than 30 min. (up to 90 min.) (duration: 0.2±0.2 s vs. control, *p* = 0.009; occurrence: 0.5±0.6 events/100 s vs. control, *p* = 0.009). After a 10–30 min. washout, evoked responses tended to recover, especially in their duration (duration: 1.0±0.5 s vs. control, *p* = 0.070; occurrence: 0.9±0.4 events/100 s vs. control, *p* = 0.011) (**Fig. 3B**). A representative recording shows the induction and progression of recovering from inhibition on the evoked depolarizations (**Fig. 3C**). After applying 1 nM Aβ42, the same recording as in the control was repeated for 30 min. in 5 min. intervals. The evoked depolarizations gradually became briefer, and finally both evoked and spontaneous depolarizations ceased at the start of washout. Following washout, evoked depolarizations re-occurred and gradually broadened. Spontaneous depolarizations also reappeared. However, there were some neurons from which the recorded responses did not fully recover, probably related to the effects of prolonged incubation in calcium-free ACSF [28], [29].

**Figure 4:**
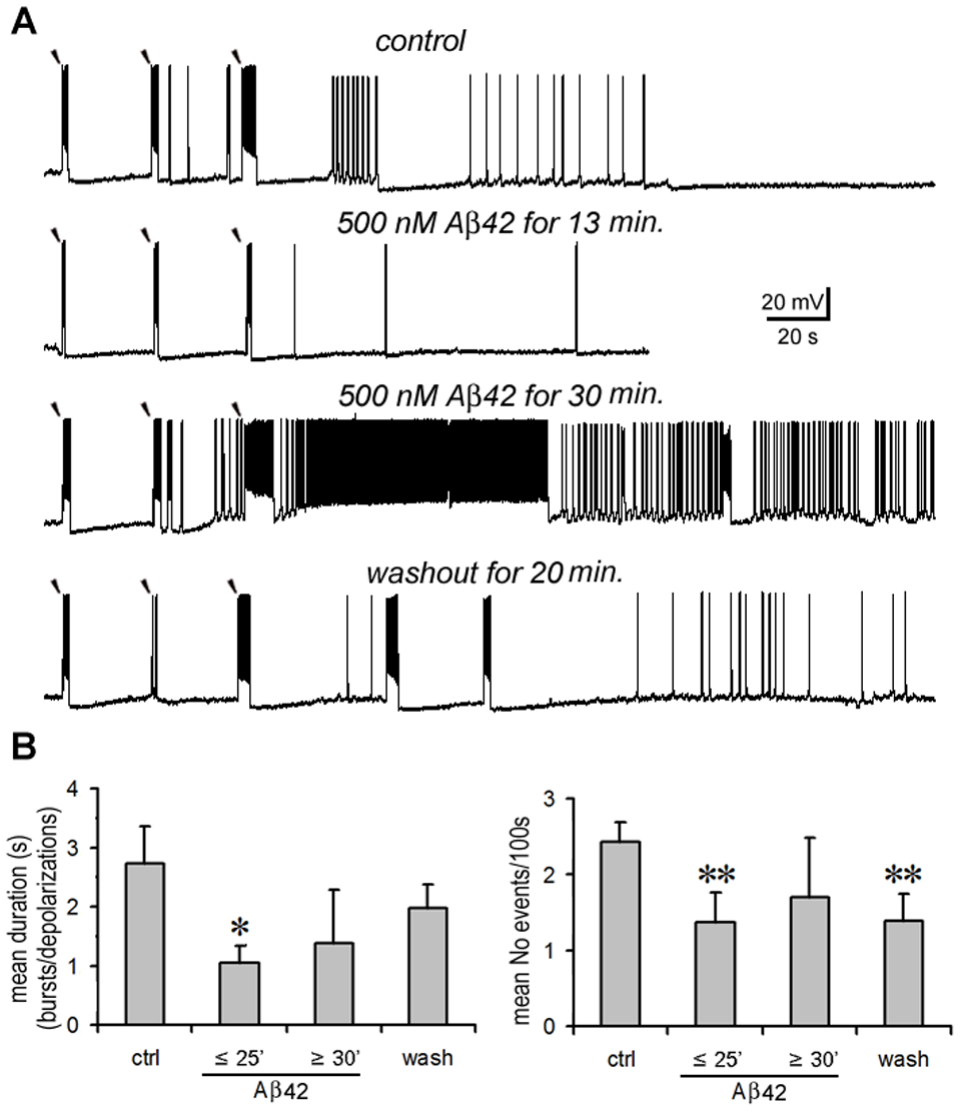
High dose Aβ42 has a biphasic effect on electrical network activities. **A.** A representative experiment. Under calcium-free conditions, a PC was recorded after delivery of three extracellular stimuli. The bursts became briefer after 500 nM Aβ42 was applied for 13 min. (2nd panel). After Aβ was applied for ≥30 min., the responses became paradoxically stronger as evidenced by the broader bursts and tonic firing that followed the 3^rd^ evoked burst (3^rd^ panel). Spontaneous responses were also enhanced (followed the 2^nd^ evoked burst in the 3rd panel). The excessive activity recovered on washout for 20 min. (lower panel). **B.** Statistical analysis: Compared with the control, the average duration (left panel) and occurrence (right panel) of evoked responses was significantly reduced after 500 nM Aβ42 was applied for ≤25 min. (duration: *p* = 0.017; occurrence: *p* = 0.002, *n* = 10, not including the cells that later showed tonic responses during prolonged application). The inhibition disappeared with Aβ applications for more than 30 min. (duration: *p* = 0.509; occurrence: *p* = 0.137). On washout, responses were similar to the control (duration: *p* = 0.332) but appeared less frequently (occurrence: *p* = 0.006). Note: “*”, *p*<0.05; “**”, *p*<0.01.

**Figure 5:**
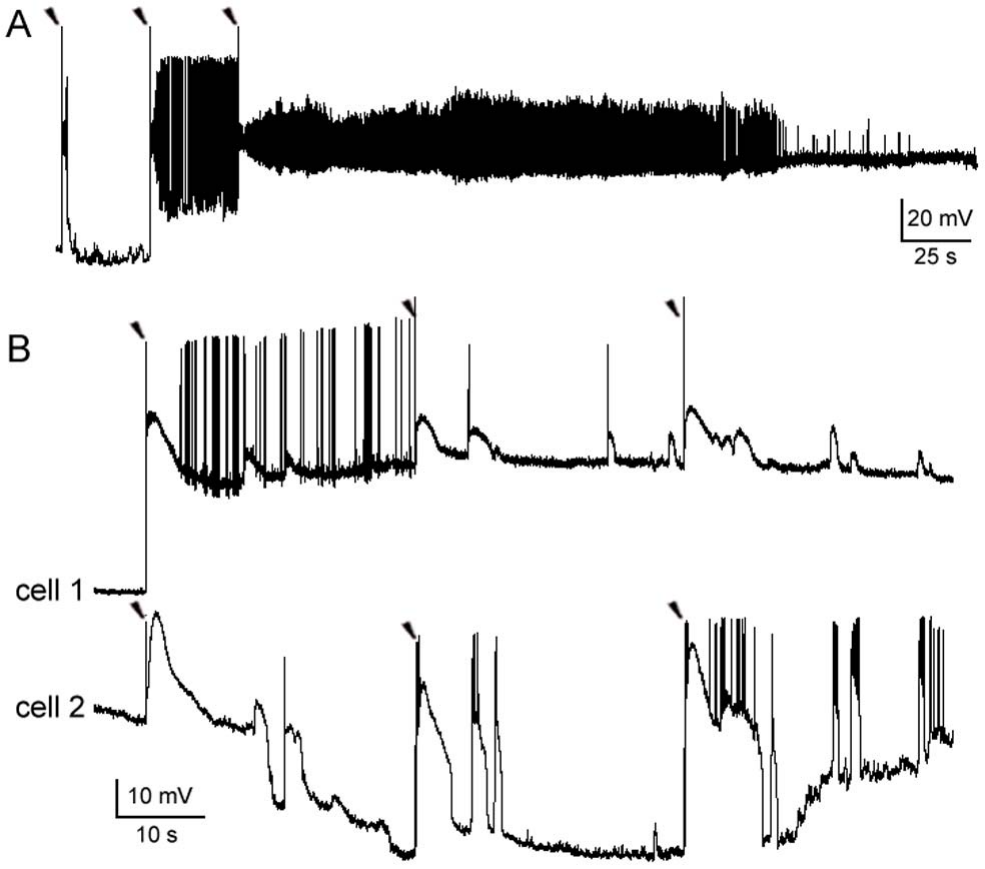
Tonic firing and supra-synchronization. **A.** A tonic firing recorded from a PC. After 500 nM Aβ42 was applied for more than 30 min., tonic firing was induced in a PC immediately after the 2^nd^ stimulus was delivered. The membrane potential thereafter was found to be depolarized to −20 mV for more than 10 min. (200 s recording was shown). **B.** Synchronization during and after tonic firing. In a PC-pair recording exposed to 500 nM Aβ42 for 30 min., a stimulating pulse in cell 1 initially provoked tonic AP firing. Its membrane potential became depolarized similar to **A**. This highly depolarized state remained stable without AP firing for more than 10 min. (not show all). Cell 2 initially experienced hyperpolarization. Nevertheless, almost all depolarizing responses, lasting for up to 10 s, showed synchronization between the two neurons.

In contrast, high doses of Aβ42 had a biphasic effect on electrical network activities according to the time window of application (**Fig. 4**). When 500 nM Aβ42 was applied for less than 25 min, the evoked responses were also significantly inhibited in terms of their duration (Aβ: 1.0±0.3 s vs. control: 2.7±0.6 s, *p* = 0.017, *n* = 10) and occurrence (Aβ: 1.4±0.4 events/100 s vs. control: 2.4±0.3 events/100 s, *p* = 0.002) (**Fig. 4A**, the 2nd trace, and **Fig. 4B**). However, when 500 nM Aβ42 was applied for more than 30 min., both evoked and spontaneous responses became paradoxically enhanced, often resulting in tonic firing (**Fig. 4A**, the 3rd trace). The mean duration and occurrence of evoked events are graphically quantified in **figure 4B**. Due to this paradoxical excitation associated with prolonged application, the inhibition of evoked responses seen with short exposure lost significance (duration: 1.4±0.9 s vs. control, *p* = 0.509; occurrence: 1.7±0.8 events/100 s vs. control, *p* = 0.137) (**Fig. 4B**). This was obviously different from the case of 1 nM Aβ42 where the inhibition became stronger in a similar prolonged time course of application (**Fig. 3B**). On washout the occurrence rate of responses also did not recover as well as in the case of 1 nM Aβ42 (**Fig. 4B right panel** vs. **Fig. 3B** **lower panel**). Furthermore, in 5 out of 15 recorded neurons (n = 5/15), tonic firing was induced that often lasted for ≥10 min. and was followed by the intensive firing of APs and/or a notable depolarization shift of membrane potentials (**Fig. 4A**, the 3^rd^ trace and **Fig. 5A**). This stands in contrast to the case of low dose Aβ42, in which no tonic firing was induced in any of the 16 recorded neurons at the later time window (n = 0/16, Chi-square test, *p* = 0.012). In one pair-recording, ‘hyper-synchronized’ responses were obtained during such a tonic response period (**Fig. 5B**). Following the stimulating pulse, one neuron (cell1) displayed a tonic AP firing and a lasting depolarization shift of its membrane potential. It later tended toward an absence of AP firing. During this period, the other neuron (cell 2) became hyperpolarized yet still exhibited bursts and depolarizations that were highly synchronous with cell 1. In summation, prolonged applications of high dose Aβ42 are likely neurotoxic. The induction of these tonic behaviors suggest that high local accumulation of Aβ42 can result in the hyper-synchronization of a local neuronal network.

**Figure 6:**
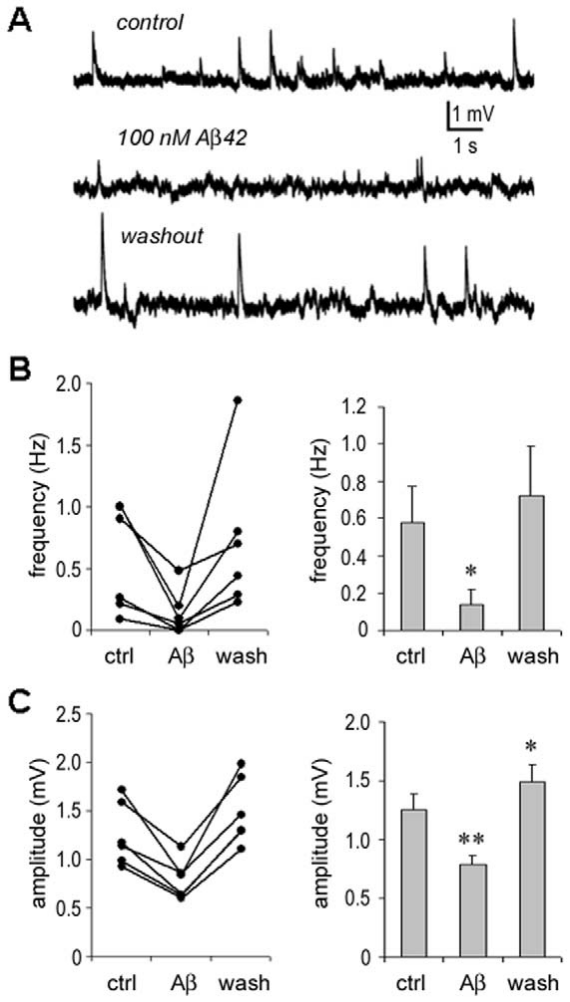
Inhibition of spontaneous subthreshold responses by Aβ42. **A.** Under calcium-free conditions, spontaneous subthreshold responses were recorded from a PC (upper panel). Responses were notably inhibited when 100 nM Aβ42 was applied for 10 min. (middle panel), and recovered following a 10 min. washout (lower panel). **B.** Statistical analysis: The frequency of spontaneous subthreshold activity was significantly reduced by applying intermediate doses of Aβ42 (*p = *0.025, *n* = 6) and recovered on washout (*p = *0.445). **C.** Statistical analysis: The amplitude of spontaneous responses was significantly reduced by applying Aβ42 (*p = *0.004) and increased on washout (*p = *0.001). Note: “*”, *p*<0.05; “**”, *p*<0.01.

In another set of experiments, effects of intermediate doses of Aβ42 on spontaneous subthreshold responses of individual neurons were examined. The responses included spikelet-like potentiations and brief depolarizations, which were captured by recording single neurons in the absence of any stimulation under calcium-free conditions (**Fig. 6A**). At Aβ42 concentrations of 10 to 100 nM for ≤10 min, spontaneous subthreshold responses occurred significantly less frequently (Aβ: 0.14±0.08 Hz vs. control: 0.58±0.17 Hz, *p* = 0.025; *n* = 6) (**Fig. 6B**) and their amplitudes were significantly reduced (Aβ: 0.78±0.08 mV vs. control: 1.26±0.13 mV, *p* = 0.004) (**Fig. 6C**). On washout of Aβ for ≤10 min., spontaneous subthreshold responses recovered (frequency: 0.72±0.25 Hz vs. control, *p* = 0.445). Moreover, the average amplitude of responses (1.49±0.14 mV) was higher than the control. The inhibition of spontaneous subthreshold responses is consistent with the low dose (and high dose but short applications) Aβ42-induced decrease in the occurrence of post-stimulus bursts and depolarizations (**Figs. 2** **–** [Fig pone-0008366-g003] **4**).

### Opposite Effects of Low and High Doses of Aβ42 on Neuronal Excitability

Neuronal excitability is an essential determinate of electrical network activities [25]. Field burst behavior, for instance, requires an increase in basal excitability [24]. AP-threshold, after-hyperpolarization (AHP) and resting membrane potential are crucial parameters that set the level of neuronal excitability. An increase in AP-threshold (depolarization) leads to a reduction of neuronal excitability, whereas its reduction (hyperpolarization) leads to an enhancement of excitability. AHP is induced following an AP or a tonic AP firing [30]. The AHP normally prevents continuous firing after a burst, contributing to rhythmic neuronal activities [31].

**Figure 7:**
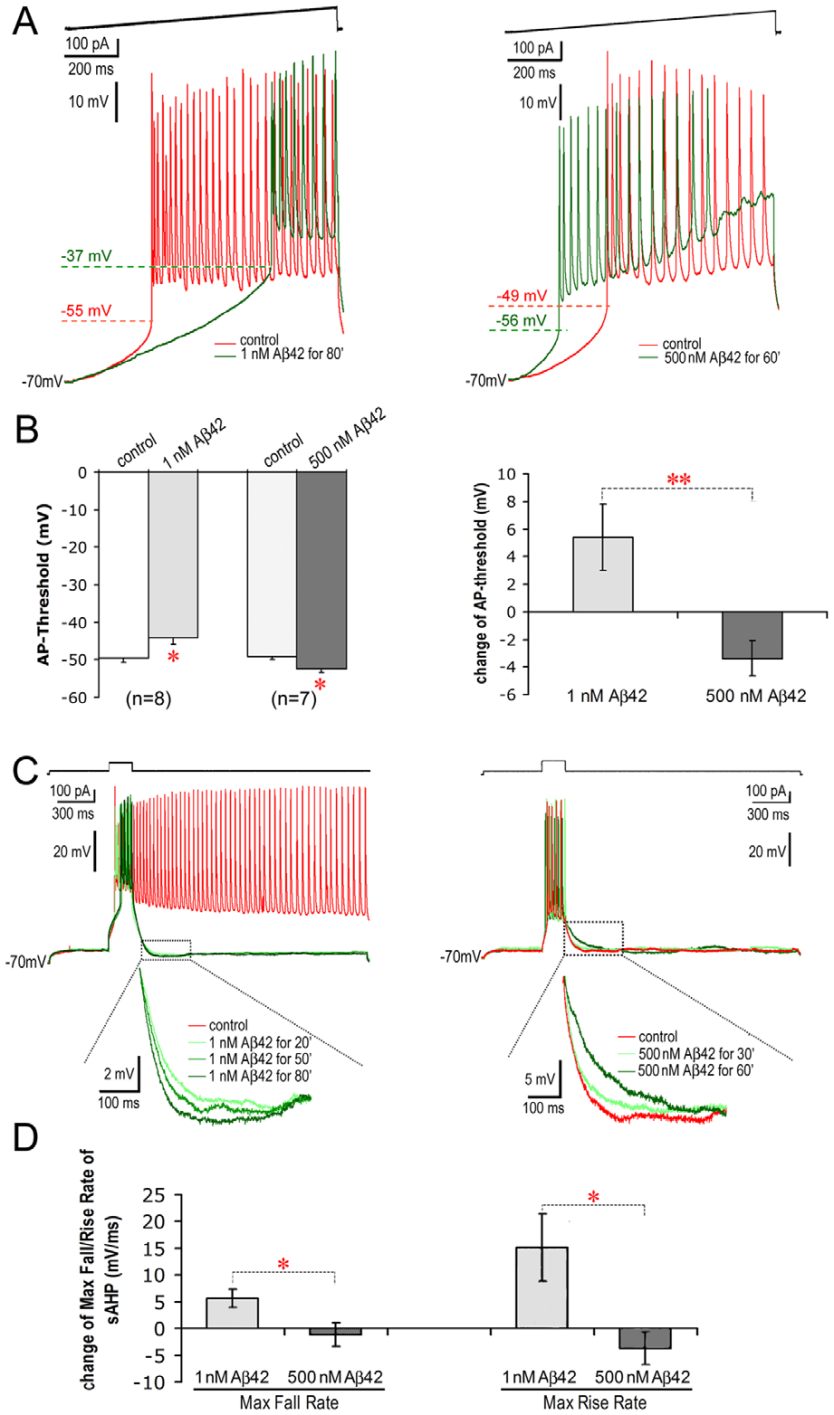
Opposing effects on AP-threshold and AHP by prolonged applications of low vs. high doses of Aβ42. **A.** AP-threshold. Under calcium-free conditions, AP-threshold of a neuron was recorded during injection of a ramp-current (above the AP traces) into the soma. After 1 nM Aβ42 was applied for 80 min. the AP-threshold of a PC increased from −55 mV to −37 mV (left panel). After 500 nM Aβ42 applications for 60 min. the AP-threshold of another PC was reduced from −49 mV to −56 mV (right panel). **B.** Statistical comparison of changes in AP-threshold. **Left Panel:** After applications of 1 nM Aβ42 for ≥30 min., the AP-threshold was significantly increased (*p* = 0.047, *n* = 8). After applications of 500 nM Aβ42 for ≥30 min., the AP-threshold was significantly reduced (*p = *0.029, *n* = 7). **Right Panel:** Opposing net changes (i.e., Aβ–control) in AP-threshold between 1 nM and 500 nM Aβ42 treatments (*p* = 0.006). **C.** AHP. **Left Panel:** After a burst induced by a depolarizing current injection for 200 ms, a tonic firing was extended in a PC. This tonic firing was attenuated by the application of 1 nM Aβ42 (green traces). Moreover, the AHP of the PC gradually deepened over time (left panel, inset). **Right Panel:** Under high dose Aβ42 exposure (500 nM), the AHP of another PC became gradually shallower over time (right panel, inset). This increase in excitability can result in tonic firing in some neurons (see figure S3). **D.** Statistical comparison of net changes in AHP. Compared with control, 1 nM Aβ42 deepened the AHP by speeding up its net maximum fall and rise rates. In contrast, 500 nM Aβ42 slowed down maximum fall and rise rates (net change in the Max Fall Rate: *p* = 0.021; net change in the Max Rise Rate: *p* = 0.025). Note: “*”, *p*<0.05; “**”, *p*<0.01.

In view of the opposite effects on electrical network activities by low and high dose Aβ42 when applied for more than 30 min., we examined the effects of these exposures on intrinsic membrane properties of neurons (**Table 1**). Significant changes were found in AP-threshold and AHP (**Fig. 7** and **Table 1**). After 1 nM Aβ42 was applied for more than 30 min., the AP-threshold was significantly increased from −49.5±1.3 mV to −44.1±1.9 mV (*p* = 0.047, *n* = 8). In contrast, after 500 nM Aβ42 was applied for more than 30 min., the AP-threshold was significantly reduced from −49.1±1.0 mV to −52.5±1.0 mV (*p* = 0.029, *n* = 7). Net changes in the two cases were opposite and statistically different (1 nM Aβ42: 5.4±2.4 mV vs. 500 nM Aβ42: −3.4±1.3 mV, *p* = 0.006, **Fig. 7A & B**). Similarly, Aβ42 at 1 nM deepened the AHP through increases in maximum fall rate by 5.6±1.7 mV/ms and maximum rise rate by 15.2±6.4 mV/ms compared with control; in contrast, Aβ at 500 nM shallowed the AHP through reductions in maximum fall rate by −2.9±1.2 mV/ms and maximum rise rate by −6.0±2.0 mV/ms (**Fig. 7C&D**). Net changes in the two cases were opposite and statistically different (in the maximum fall rate: *p* = 0.021; in the maximum rise rate: *p* = 0.025; **Fig. 7D**). The combined deepening of AHP and raising of AP-threshold probably contributed to make neurons less excitable. For example, the deepened AHP in the presence of low dose Aβ42 was associated with the termination of extended firing (**Fig. 7C left panel**). In contrast, the shallowed AHP and reduced AP-threshold in the case of high dose Aβ42 probably contributed to make the neurons more excitable. This property likely contributed to the occurrence of extended tonic firing (**Fig. 7C right panel** **& Fig. S3**). These opposing changes in neuronal excitability match the aforementioned observations pertaining to electrical network activities.

**Table 1:** Changes in intrinsic membrane properties of PCs by prolonged application of low and high dose Aβ42.

	Aβ42 (1 nM, *n* = 8)		Aβ42 (500 nM, *n* = 7)	
	ctrl	Aβ	ctrl	Aβ
Resting membrane potential (mV)	−64.6±2.1	−61.5±2.4	−66.7±2.5	−66.0±2.8
**AP analysis**				
Threshold (mV)	−49.4±1.3	−44.1±1.9*	−49.1±1.0	−52.5±1.0*
AP_amplitude (mV)	62.5±9.8	55.4±9.2	68.9±5.8	60.7±11.2
AP_half_duration (ms)	2.1±0.3	2.3±0.4	2.2±0.1	1.8±0.4
AP_rise_rate (mV/ms)	39.4±6.1	35.9±6.6	45.1±4.6	38.8±8.0
**AHP Analysis**				
Max_fall_rate (mV/ms)	−11.4±2.8	−17.0±2.1$	−21.1±5.0	−20.0±5.0$
Max_rise_rate (mV/ms)	32.2±6.1	47.3±4.6$	51.5±9.2	47.8±9.1$

* *p*<0.05.

$ Net change (Aβ - ctrl) is statistically significant (*p*<0.05; see Fig. 7D).

Note 1: During application of Aβ, AP and AHP were recorded while clamping membrane potential at the same level as control.

Note 2: Data are mean±SE. (For the data of mean±SD, please see Table S2).

## Discussion

We undertook this study in PFC because it is affected pathologically with Aβ early in AD and is the seat of working memory. Working memory is a brain function that is considerably reliant on neuronal synchronization [3]. Patients with AD showed reductions in neuronal synchronization during the performance of working memory task [32]. It has been puzzling that changes in AD appear to be opposing to include inhibited neuronal synchronization [4] and supra-synchronization [5]. This phenomenon in AD progression is a paradigm for the opposing actions of Aβ42 on electrical network activities that we report. The major finding of our study is that opposite changes in electrical network activities were induced by low and high doses of Aβ42. These could be explained by corresponding opposite changes in the excitability of individual neurons. Electrical network activities and neuronal synchronization were significantly inhibited by low dose Aβ42 and initially for short applications only, by high dose Aβ42. However, longer applications of high dose Aβ42 significantly enhanced electrical network activities and synchrony between neurons, even resulting in tonic behaviors in individual neurons. Moreover, applications of low and high doses of Aβ42 induced opposite changes in AP-threshold and AHP. Low dose Aβ42 significantly increased the AP-threshold and deepened the AHP, making neurons less excitable. High dose Aβ42 significantly reduced the AP-threshold and shallowed the AHP, making neurons more excitable.

### Implications for the Biological Function of Aβ

Aβ is considered to have physiological functions in part because it is normally present in cerebrospinal fluid at low levels [33], [34], [35]. In recent years, it has been found that the neuronal secretion of Aβ by exocytosis depends on the activity of excitatory chemical synaptic transmission [36], [37], [38]. In turn, Aβ has a postulated feedback-inhibitory role to maintain homeostasis in neurotransmitter-based networks [37], [38]. Aβ modifies chemical synaptic structure and function by promoting the endocytosis of synaptic AMPA and NMDA receptors [38], [39]. This postulated normal biologic function of Aβ is supported by evidence that rodent Aβ, while unable to form pathologic aggregates, still depresses excitatory chemical synaptic transmissions [37]. Conversely, the absence of Aβ production in amyloid precursor protein (APP) knockout mice leads to hyperexcitability in neuronal circuits and lowered threshold to kainite-induced seizure activity [40].

Field bursting behaviors under either of low calcium-containing *in vivo* or *in vitro* conditions, are interpreted as arising from electrical-based interactions over networks and have been demonstrated in both physiologic and pathologic states (for a review, see [25]). Their induction depends on the synchronization of responses involving many individual pyramidal neurons [24], [27]. The same phenomenon recorded between individual pyramidal neurons in the current study, albeit on a much smaller scale, represents similar synchronous behavior between neurons belonging to prefrontal cortical electrical networks.

Our study is the first, to our knowledge, to examine how the Aβ peptide might regulate the activity of an electrical network. We show stable inhibition when Aβ42 is applied at low concentrations that approximate physiological levels [33], [34], [35]. Therefore, Aβ could have a constraining biological function, not just on chemical synaptic transmissions but also on electrical network activities. In view of this finding, the hypersensitivity to seizures reported in the APP knockout mice [40] could be due to deficits in feedback inhibition by Aβ at the level of electrical networks, as well as of chemical synaptic transmissions. However, a recent study raised questions on the postulated Aβ-feedback inhibition because picomolar Aβ was found to positively modulate chemical synaptic plasticity and memory [41]. If this holds true, the Aβ-inhibition of electrical network activity could assume added importance as perhaps the only feedback mechanism to balance the homeostatsis of neuronal networks and hence to constrain the secretion of Aβ as well.

### Dyshomeostatsis in Neuronal Networks

In AD, the accumulation of Aβ, especially Aβ42, is a major pathological change [7], [8], [36] and the abnormality in neuronal synchronization is well described [4], [5]. Notably, there are no previous reports showing direct evidence that Aβ inhibits neuronal synchrony. Instead, many studies provide indirect evidence based on impairments of chemical synaptic transmissions by Aβ [7], [13], [38]. For instance, Aβ42 at high nanomolar concentrations, especially oligomeric preparations, impair various synaptic functions such as the induction of long-term potentiation (LTP, a form of associative memory) [10], [38]. Our study provides direct evidence that low Aβ42 levels inhibit the synchronization between individual neurons, and the inhibition becomes stronger as the exposure is prolonged. The latter case may mimic conditions in early stages of AD, during which Aβ peptides may not be cleared promptly after secretion and then accumulate. The strengthened inhibition of synchronicity induced by Aβ accumulation may account for some cognitive decline in early AD. However, inhibiting actions of Aβ cannot explain the paradoxical over-excitation of neuronal networks - another phenomenon found in patients and in up to 65% of mice carrying human AD-causing mutations [5], [42]. Our study also provides a clue to the explanation of this phenomenon.

Two factors considered crucial to the conversion from inhibition to excitation are the applied concentration and duration of Aβ. We found a biphasic action of Aβ42 at higher concentrations, which consists of an initial inhibition followed with prolonged application by the reappearance and then exaggeration of synchronicity. Thus, high concentration Aβ42 for prolonged periods of time led to excitation and even tonic firing in one third of the recorded neurons. It is therefore possible that an accumulation of high-levels of Aβ42 in some extracellular regions could induce an over-excitation of neuronal networks. Together with the tendency toward hyper-synchrony, seizure activity to an extent as documented in AD patients and animal models may result. Consistent with this hypothesis, seizures tend to become more frequent toward the late stages of AD [40], [43]. Abnormal levels of neuronal activity such as tonic firing could additively lead to neurotoxicity [44]. While the latter is expected to ultimately suppress the activity of some neurons and their networks, epileptic activity in others may increase. Taken together, high levels of Aβ42 accumulation can have an added polar role: the suppression of activity and plasticity in neurotransmitter-based excitatory synaptic networks while enhancing the activities of electrical networks. This would help to explain the coexistence of over-excitation in cortical and hippocampal circuits and aberrant sensitivity to Pentylenetetrazol (PTZ)-induced seizures with impairments in glutamatergic excitatory synaptic transmissions in the hAPP FAD mice [5].

### Underlying Mechanisms

Intrinsic membrane properties of neurons are essential to the activation state of electrical networks [24]. Interactions between electrical synaptic and intrinsic membrane properties produce neuronal synchronizing phenomena such as oscillatory network activity [45]. Computer simulations also reveal that neuronal excitability is an essential factor determining neuronal synchrony [46]. In a recent study using brain slices from monocular deprived rats, enhanced neuronal excitability was found to overcome reductions in chemical synaptic transmissions resulting in a net stimulation of network activity [47]. Therefore, the influence of Aβ42 on neuronal excitability could be sufficient to lead the activity state of whole networks. Changes in intrinsic membrane properties by low and high doses of Aβ42 may serve, at least in part, as the cellular basis for the opposing effects on electrical network activities and neuronal synchronization. According to ours and previous studies [34], [48], low physiologic levels of Aβ may be necessary to maintain a cap on neuronal excitability by modulating intrinsic membrane properties such as AP-threshold and AHP. In this way, Aβ stabilizes electrical networks and chemical synaptic transmissions after input signals. Following rapid clearance, neuronal networks recover and are ready for subsequent inputs. Under advancing AD conditions, we hypothesize that networks become more excitable due to enhanced electrical network activities arising from changes such as reduced AP-thresholds and shallowed AHPs. Tonic firing, supra-synchronization and epileptiform activity become more common. Consistent with this, hyperexcitability of pyramidal cells was reported in an AD transgenic mouse model that exhibited seizure behaviors [42].

In terms of possible molecular mechanisms, we speculate that Aβ42 could act in a time- and concentration-dependent manner on the ion channels that underlie AP-threshold and AHP. Aβ42 may influence certain biophysical properties of these ion channels such as open time and conductance, as well as their internalization or expression on neuronal membranes. For instance, the AHP constitutes the post-AP repolarization phase which relies on the activation of potassium channels including calcium activated, voltage-gated and delayed-rectified potassium channels [49], [50], [51]. It has been reported that a short Aβ fragment (Aβ25–35) first increased the expression level of a voltage-gated potassium channel subtype, but after a prolonged application, the expression level decreased [52]. The opposite changes in AHP reported in current study could be induced via a similar mechanism. Biphasic effects on voltage-gated sodium channels that underlie AP-threshold levels are also possible. These topics are worthy of future study.

Another major contributor to electrical network activities and neuronal synchronization is the electrical synapse (i.e., gap junction) [53], [54], [55], [56]. Gap-junctions between PCs are distributed sparsely in the neocortex, but characterized by high junctional conductance [57], [58]. Pyramidal gap junctions hence present an extraordinary ability for signal amplification in the neocortex. For instance, an input signal, through a small set of electrically coupled PCs, can become strong enough to initiate a local field potential and hence achieve synchronicity between individual neurons. It is highly possible that the effects of Aβ on electrical network activities reported here are conveyed also via acting on gap junctions. This possibility remains to be studied in future work.

## Materials and Methods

### Slice Preparation

Wistar rats were used acutely for the purpose of obtaining and preparing brain slices. Housing and surgical procedures of animals used for recording were in accordance with the National Institutes of Health guidelines and the Tufts University Institutional Animal Care and Use Committee.

Prefrontal cortical slices (300 µm) were prepared from young (P16–P30) and adult rats (3–12 months) using a published protocol [59]. Animals were euthanized by intraperitoneal injection of sodium pentobarbital (0.2 mg/g), decapitated, and their brains immediately removed and placed in cold artificial cerebrospinal fluid (ACSF). Brain sections were cut on a vibratome and transferred into ACSF which was continuously oxygenated with 95% O_2_ and 5% CO_2_. Brain slices were incubated at 34°C for 30 minutes and then at room temperature for at least 30 minutes before recording. During recording, brain slices were maintained at 34°C in a recording chamber and perfused with oxygenated ACSF at a flow rate of 0.75–1.0 ml/min. The ACSF volume in the tube leading to and including the recording chamber was 1.5–2.0 ml. This enabled the quick replacement of recording solutions (≤3 min.) during different experimental procedures. After recording, brain slices were fixed with 2% paraformaldehyde and 1% glutaraldehyde and histochemically stained.

### Electrophysiological Recordings

Under calcium-free conditions, multi-neuron patch clamp recordings were carried out to capture synchronized responses, both spontaneous and provoked, between single neurons [60]. Somatic whole-cell recordings (6–12 MΩ pipette resistance) were made in which signals were amplified using Axoclamp-200B amplifiers (Axon Instruments, USA). Recordings were sampled using the program Igor (Igor Wavemetrics, Lake Oswego, OR, USA), digitized by an ITC-18 interface (Instrutech, Great Neck, NY, USA) and stored on a hard driver (Macintosh G5 computer) for off-line analysis (Igor). Voltages were recorded with pipettes containing (mM): 100 potassium gluconate, 20 KCl, 4 ATP-Mg, 10 phosphocreatine, 0.3 GTP, 10 Hepes (pH 7.3) and 0.4% biocytin (Sigma). The recording (bathing) solution, except for zero calcium, was typical artificial cerebrospinal fluid (ACSF), containing (mM): 125 NaCl, 2.5 KCl, 25 glucose, 25 NaHCO_3_, 1.25 NaH_2_PO_4_, and 1 MgCl_2_. Access resistance was monitored. Only the neurons with stable access resistance were included in the statistical analyses. But for the observation of synchronized responses, some neurons with increased access resistance were still included. All recordings were performed at resting membrane potentials of neurons. To deliver extracellular stimuli, glass electrodes (2–3 MΩ pipette resistance) were filled with calcium-free ACSF and placed 100–200 µm away from recorded neurons. Neurons were filled with biocytin by diffusion during recording for later identification of PCs by morphology and AP firing properties (**Fig. S4**) [61].

Spontaneous responses were recorded from individual PCs in layer 5 of the PFC using multi-neuron whole-cell patch clamp technique. Evoked bursts and depolarizations were obtained after a brief extracellular stimulus (10–100 µA for 2 ms) delivered nearby simultaneously recorded neurons. The extracellular stimulus strength was first gradually increased and was then set after a burst was provoked in at least one of the recorded neurons. Neuronal synchronization was identified according to the coordinated occurrence of responses between multiple neurons. The duration and occurrence of evoked bursts and depolarizations were analyzed using the Igor program. The frequency and amplitude of spontaneous subthreshold responses were analyzed using the software Origin (Origin Lab Corp. Wellesley Hills, MA, USA).

Intrinsic membrane properties of PCs were obtained from the recording of a large set of stimulations [62]. Somatic depolarizing current injections caused discharges ranging from 30–300 pA, depending on the individual neuronal input resistance. In order to standardize the analyzed profiles across all neurons, the stimulus strength was normalized according to the minimal step current required to reach AP-threshold (the membrane potential above which an action potential is initiated). Among the key parameters representing neuronal excitability, AP-threshold and AHP (a period of repolarization and hyperpolarization following an AP or a tonic AP firing) were chosen because their changes were significant. AP-threshold was recorded by giving a ramp-current injection into soma. AHP was induced after a burst that was evoked by a current injection of depolarizing step into soma (200 ms duration). The analysis of these properties was carried out using a specially programmed Igor experiment [62].

### Preparation and Use of Soluble Synthetic Aβ42

Synthetic Aβ42 peptide was purchased from Biosource (Camarillo, CA). Aliquots of 5 nmol Aβ42 treated by 1,1,1,3,3,3-hexafluoro-2-propanol (HFIP) were stored at −80°C [63]. A working stock of 0.1 mM Aβ42 in 5% DMSO and 25 mM Tris-HCl, pH 7.4 was prepared days before use and also stored at −80°C. The monomeric Aβ42 that was used in these experiments was monitored by western blot. The stock of Aβ42 was loaded on NuPAGE Novex 4–12% Bis-Tris gels (Invitrogen). Mouse monoclonal anti-human amyloid beta protein antibody (6E10, purchased from Signet) was used at a 1000X dilution. Horse anti-mouse IgG HRP-linked antibody (Cell Signaling Technology, 2000X dilution) was used as the secondary antibody. The chemiluminol reaction was recorded by hyperfilm (Amersham Biosciences). Aβ42 oligomers were not clearly visible on the western blots. Different concentrations of Aβ42 were freshly prepared on the day of use by defrosting and diluting the stock solution with ACSF. Soluble synthetic Aβ42 or vehicle (control medium) was bath-applied either at low doses of 1–100 nM or at high doses of 500 nM. To examine the effects of Aβ42, each recording procedure was repeated in three conditions: *a)* pre-application, *b)* application and *c)* washout of Aβ. Aβ42 was applied for periods lasting 30 to 90 min. Spontaneous subthreshold responses were recorded for up to 20 min. The Aβ-washouts were recorded for periods lasting 10 to 60 min.

### Statistical Analysis

Paired t-test was used for the comparison of a variant between different recording procedures. Student t-test was used for the comparison of a variant between different treatments of Aβ. Chi-square test was used for the comparison between two percentage values. Data were shown as mean±SE (or SD in the table S2).

## Supporting Information

Figure S1 No effect on electrical network activity by cocktail blockers of chemical synaptic transmissions. Under calcium-free conditions, spontaneous electrical network responses were recorded at the resting membrane potential of a PC, which were not influenced by the application of cocktail blockers (100 µM APV to NMDA receptors, 10 µM DNQX to AMPA receptors, 20 µM picrotoxin to GABA receptors).
(4.07 MB TIF)

Figure S2 Experimentally provoked depolarization and burst. Under calcium-free conditions, an extracellular stimulus of 20 µA with a 2 ms duration was delivered near a PC. The stimulus artifact is shown (arrowhead). Thereafter, a short depolarization (red trace) was provoked. An increase of the extracellular stimulus to 25 µA provoked a burst after the stimulating pulse.
(0.22 MB TIF)

Figure S3 Prolonged application of 500 nM Aβ42 promotes tonic firing of a neuron. Under calcium-free conditions, a brief burst firing (red trace) was induced by the current injection of a depolarizing step (50 pA for 200 ms, upper trace) into the soma of a PC. After applying 500 nM Aβ42 for 30 min., the same stimulus induced a burst followed by an extended AP firing for several hundred milliseconds (light green trace). After applying 500 nM Aβ42 for 60 min., the same stimulus induced a burst followed by tonic AP firing (dark green trace).
(2.57 MB TIF)

Figure S4 Identification of pyramidal cells that were recorded from PFC slices. A. Histochemical staining of two layer 5 PCs after recording. Two PCs in layer 5 were filled with biocytin during recording and then later stained. B. Stepped-depolarization-current injections evoked non-accommodating AP firing patterns typical for layer 5 PCs in the PFC (see Ref. Wang et al. Nat. Neurosci. 2006). The recording was performed under calcium-free conditions.
(4.20 MB TIF)

Table S1 Percentage of cells with different responses before and after stimuli.
(0.03 MB DOC)

Table S2 Changes in intrinsic membrane properties of PCs by prolonged application of low and high dose Aβ42. Note 1: During application of Aβ, AP and AHP were recorded while clamping membrane potential at the same level as control. Note 2: Data are mean±SD. Note 3: The AHP notably varied among the recorded neurons, ranging from 14 mV/ms to 75 mV/ms in the max fall rate and from −5 mV/ms to −36 mV/ms in the max rise rate. The difference in the max fall/rise rates of the AHP did not reach statistical significcance between the two groups in the control condition (For the max rise rate: p = 0.089; For the max fall rate: p = 0.223).
(0.04 MB DOC)
