# Supplementary figures and images for: Opposite Effects of Low and High Doses of Aβ42 on Electrical Network and Neuronal Excitability in the Rat Prefrontal Cortex
Source: PLoS One. 2009 Dec 21;4(12):e8366. doi: 10.1371/journal.pone.0008366 (PMC2791225; doi:10.1371/journal.pone.0008366)

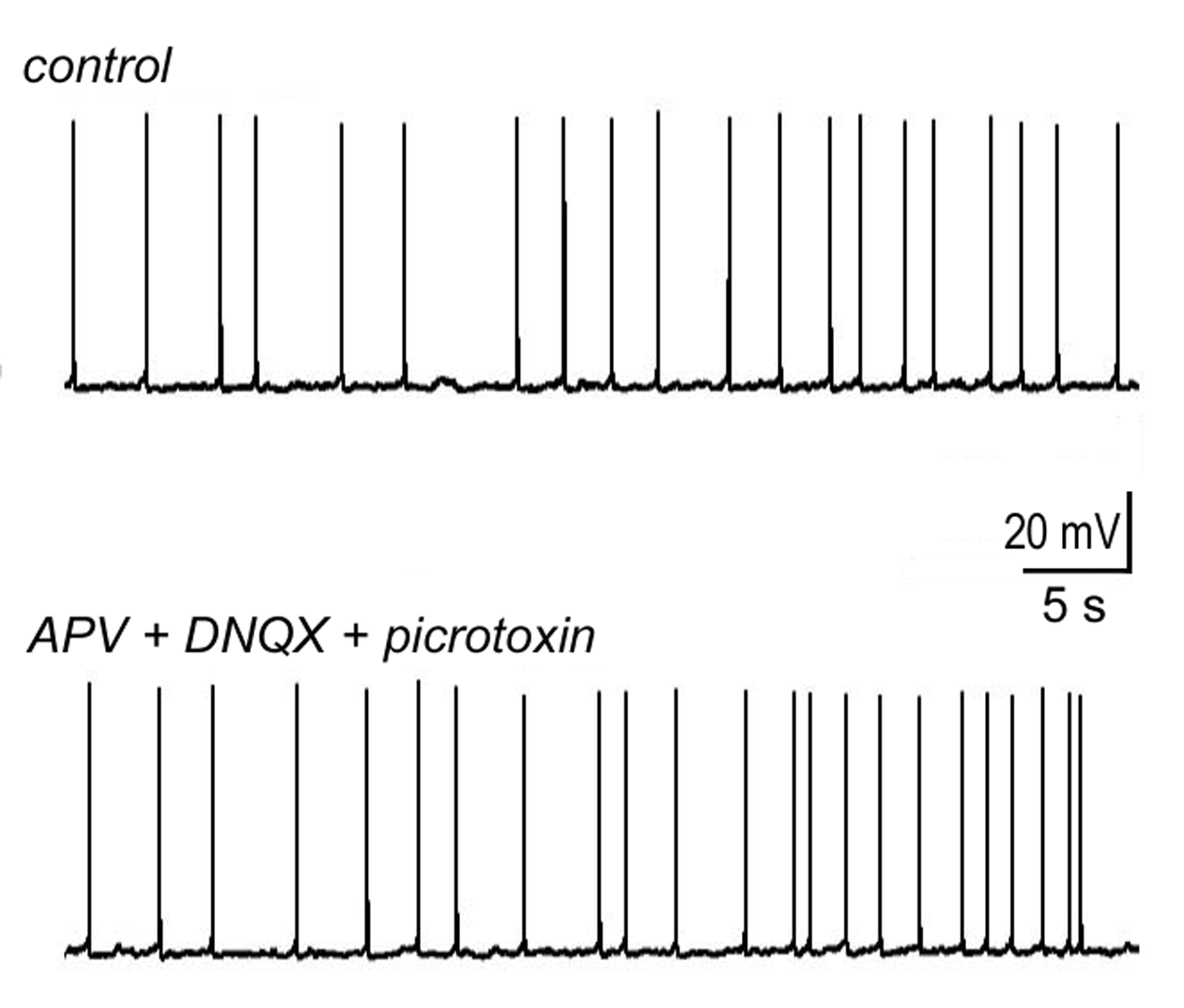

Supplement: Figure S1 — No effect on electrical network activity by cocktail blockers of chemical synaptic transmissions. Under calcium-free conditions, spontaneous electrical network responses were recorded at the resting membrane potential of a PC, which were not influenced by the application of cocktail blockers (100 µM APV to NMDA receptors, 10 µM DNQX to AMPA receptors, 20 µM picrotoxin to GABA receptors). (4.07 MB TIF) [file pone.0008366.s001.tif]

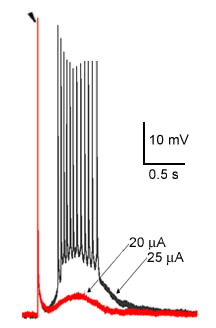

Supplement: Figure S2 — Experimentally provoked depolarization and burst. Under calcium-free conditions, an extracellular stimulus of 20 µA with a 2 ms duration was delivered near a PC. The stimulus artifact is shown (arrowhead). Thereafter, a short depolarization (red trace) was provoked. An increase of the extracellular stimulus to 25 µA provoked a burst after the stimulating pulse. (0.22 MB TIF) [file pone.0008366.s002.tif]

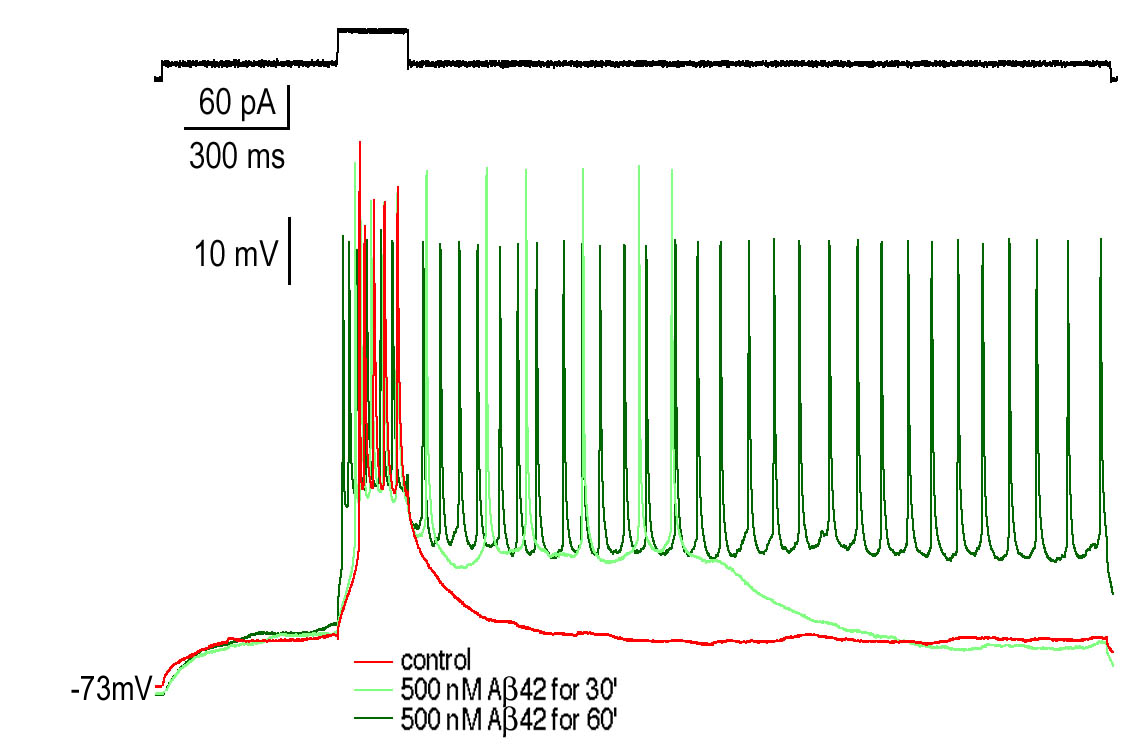

Supplement: Figure S3 — Prolonged application of 500 nM Aβ42 promotes tonic firing of a neuron. Under calcium-free conditions, a brief burst firing (red trace) was induced by the current injection of a depolarizing step (50 pA for 200 ms, upper trace) into the soma of a PC. After applying 500 nM Aβ42 for 30 min., the same stimulus induced a burst followed by an extended AP firing for several hundred milliseconds (light green trace). After applying 500 nM Aβ42 for 60 min., the same stimulus induced a burst followed by tonic AP firing (dark green trace). (2.57 MB TIF) [file pone.0008366.s003.tif]

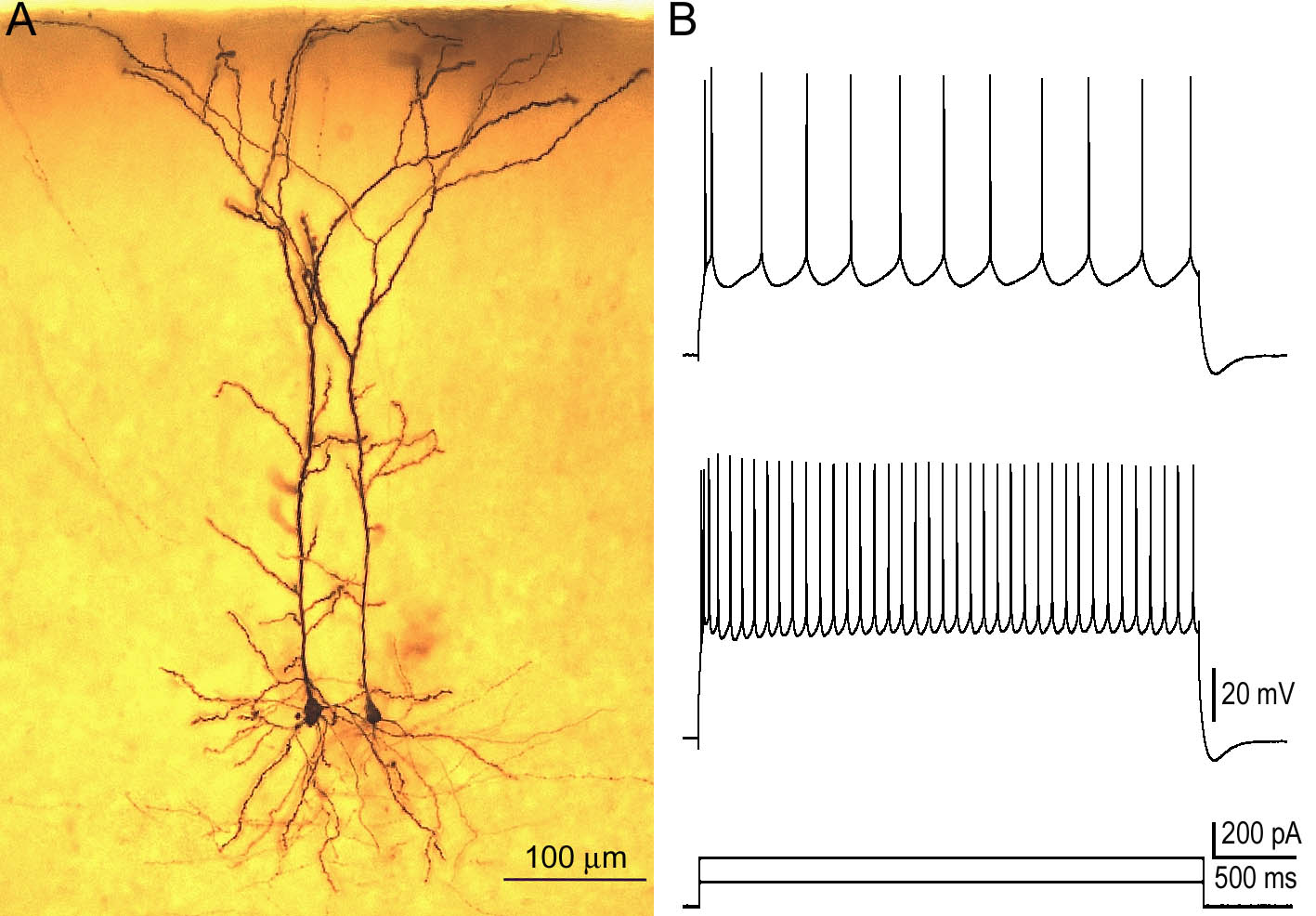

Supplement: Figure S4 — Identification of pyramidal cells that were recorded from PFC slices. A. Histochemical staining of two layer 5 PCs after recording. Two PCs in layer 5 were filled with biocytin during recording and then later stained. B. Stepped-depolarization-current injections evoked non-accommodating AP firing patterns typical for layer 5 PCs in the PFC (see Ref. Wang et al. Nat. Neurosci. 2006). The recording was performed under calcium-free conditions. (4.20 MB TIF) [file pone.0008366.s004.tif]
