# Supplementary material for: Opposite Effects of Low and High Doses of Aβ42 on Electrical Network and Neuronal Excitability in the Rat Prefrontal Cortex
Source: PLoS One. 2009 Dec 21;4(12):e8366. doi: 10.1371/journal.pone.0008366 (PMC2791225; doi:10.1371/journal.pone.0008366)
Supplement: Table S1 — Percentage of cells with different responses before and after stimuli. (0.03 MB DOC) [file pone.0008366.s005.doc]

| | **Table S1: Percentage of cells with different responses before and after stimuli** | | | | | --- | --- | --- | --- | |  | AP | depolarization/burst | tonic firing | | before stimuli | 18% | 18% | 0% | | after stimuli | 22% | 43% | 10% | | *p* = | 0.143 | 0.009 | 0.021 | | n = 49 cells; |  |  |  | | Compared using Chi-square test. | |  |  | |
| --- | --- | --- | --- | --- | --- | --- | --- | --- | --- | --- | --- | --- | --- | --- | --- | --- | --- | --- | --- | --- | --- | --- | --- | --- | --- | --- | --- | --- |
