# Supplementary material for: Opposite Effects of Low and High Doses of Aβ42 on Electrical Network and Neuronal Excitability in the Rat Prefrontal Cortex
Source: PLoS One. 2009 Dec 21;4(12):e8366. doi: 10.1371/journal.pone.0008366 (PMC2791225; doi:10.1371/journal.pone.0008366)
Supplement: Table S2 — Changes in intrinsic membrane properties of PCs by prolonged application of low and high dose Aβ42. Note 1: During application of Aβ, AP and AHP were recorded while clamping membrane potential at the same level as control. Note 2: Data are mean±SD. Note 3: The AHP notably varied among the recorded neurons, ranging from 14 mV/ms to 75 mV/ms in the max fall rate and from −5 mV/ms to −36 mV/ms in the max rise rate. The difference in the max fall/rise rates of the AHP did not reach statistical significcance between the two groups in the control condition (For the max rise rate: p = 0.089; For the max fall rate: p = 0.223). (0.04 MB DOC) [file pone.0008366.s006.doc]

| | **Table S2: Changes in intrinsic membrane properties of PCs by prolonged application of low and high dose Aβ42** | | | | | | | | | | | --- | --- | --- | --- | --- | --- | --- | --- | --- | --- | |  | **Aβ42** (1 nM, *n* = 8) | | | |  | | **Aβ42** (500 nM, *n* = 7) | | | |  | **ctrl** | | **Aβ** | |  | | **ctrl** | | **Aβ** | | Resting membrane potential (mV) | -65 ± 6 | | -66 ± 6 | |  | | -67 ± 6 | | -66 ± 3 | | **AP analysis** |  | |  | |  | |  | |  | | Threshold (mV) | -49 ± 4 | | -44 ± 5* | |  | | -49 ± 2 | | -52 ± 2* | | AP_amplitude (mV) | 63 ± 26 | | 55 ± 24 | |  | | 69 ± 14 | | 61 ± 28 | | AP_half_duration (ms) | 2.1 ± 0.8 | | 2.3 ± 1.1 | |  | | 2.2 ± 0.4 | | 1.8 ± 0.9 | | AP_rise_rate (mV/ms) | 39 ± 16 | | 36 ± 17 | |  | | 45 ± 11 | | 39 ± 20 | | **AHP Analysis** |  | |  | |  | |  | |  | | Max_fall_rate (mV/ms) | -11 ± 6 | | -17 ± 5^$^ | |  | | -21 ± 11 | | -20 ± 11^$^ | | Max_rise_rate (mV/ms) | 32 ± 14 | | 47 ± 10 ^$^ | |  | | 51 ± 21 | | 48 ± 20^$^ | | * *p* < 0.05. |  | |  | |  | |  | |  | | ^$^ Net change (Aβ - ctrl) is statistically significant (*p* < 0.05; see Fig. 7D). | | | | | |  | | |  | | Note 1: During application of Aβ, AP and AHP were recorded while clamping membrane potential at the same level as control. | | | | | | | | | | | Note 2: Data are mean ± SD. | |  | |  | |  | |  |  | |
| --- | --- | --- | --- | --- | --- | --- | --- | --- | --- | --- | --- | --- | --- | --- | --- | --- | --- | --- | --- | --- | --- | --- | --- | --- | --- | --- | --- | --- | --- | --- | --- | --- | --- | --- | --- | --- | --- | --- | --- | --- | --- | --- | --- | --- | --- | --- | --- | --- | --- | --- | --- | --- | --- | --- | --- | --- | --- | --- | --- | --- | --- | --- | --- | --- | --- | --- | --- | --- | --- | --- | --- | --- | --- | --- | --- | --- | --- | --- | --- | --- | --- | --- | --- | --- | --- | --- | --- | --- | --- | --- | --- | --- | --- | --- | --- | --- | --- | --- | --- | --- | --- | --- | --- | --- | --- | --- | --- | --- | --- | --- | --- | --- | --- | --- | --- | --- | --- | --- | --- | --- | --- | --- | --- | --- | --- | --- | --- | --- | --- | --- | --- | --- | --- | --- | --- | --- | --- | --- | --- | --- | --- | --- | --- | --- | --- | --- | --- | --- | --- | --- | --- | --- | --- | --- | --- | --- | --- | --- | --- | --- |
